# The prevalence of headache disorders in children and adolescents in Ethiopia: a schools-based study

**DOI:** 10.1186/s10194-020-01179-2

**Published:** 2020-09-01

**Authors:** Yared Zenebe Zewde, Mehila Zebenigus, Hanna Demissie, Redda Tekle-Haimanot, Derya Uluduz, Tayyar Şaşmaz, Fatma Bozdag, Timothy J. Steiner

**Affiliations:** 1 Department of Neurology, School of Medicine, College of Health Sciences, Addis Ababa University, Addis Ababa, Ethiopia; 2 Department of Internal Medicine, School of Medicine, College of Health Science, Addis Ababa University, Addis Ababa, Ethiopia; 3 Neurology Department, Cerrahpaşa School of Medicine, Istanbul University, Istanbul, Turkey; 4 Public Health Department, School of Medicine, Mersin University, Mersin, Turkey; 5 Department of Neuromedicine and Movement Science, Norwegian University of Science and Technology, Edvard Griegs gate, Trondheim, Norway; 6 Division of Brain Sciences, Imperial College London, London, UK

**Keywords:** Child and adolescent headache, Migraine, Tension-type headache, Medication-overuse headache, Undifferentiated headache, Epidemiology, Prevalence, Schools-based study, Ethiopia, Global campaign against headache

## Abstract

**Background:**

The Global Burden of Disease (GBD) study establishes headache as the second-highest cause of disability worldwide. Because most headache data in GBD are from adults, leading to underestimation of headache-attributed burden, a global schools-based programme within the Global Campaign against Headache is contributing data from children (7–11 years) and adolescents (12–17 years). This national study in Ethiopia is the first in this programme reported from sub-Saharan Africa.

**Methods:**

A cross-sectional survey following the generic protocol for the global study was conducted in six schools (urban and rural), in Addis Ababa city and three regions of Ethiopia. Structured questionnaires were self-completed under supervision by pupils within their classes. Headache diagnostic questions were based on ICHD-3 beta criteria but for the inclusion of undifferentiated headache (UdH).

**Results:**

Of 2349 potential participants, 2344 completed the questionnaire (1011 children [43.1%], 1333 adolescents [56.9%]; 1157 males [49.4%], 1187 females [50.6%]), a participation proportion of 99.8%. Gender- and age-adjusted 1-year prevalence of headache was 72.8% (migraine: 38.6%; tension-type headache: 19.9%; UdH: 12.3%; all headache on ≥15 days/month: 1.2%; probable medication-overuse headache: 0.2%). Headache was more prevalent in females (76.2%) than males (71.0%), a finding reflected only in migraine among the headache types. Headache was more prevalent among adolescents (77.6%) than children (68.4%), reflected in all types except migraine, although prevalence of UdH fell sharply after age 14 years to 3.9%.

For headache overall, findings matched those in Turkey and Austria, obtained with the same questionnaire, but the high prevalence of migraine, not increasing with age, was surprising. The study highlighted diagnostic difficulties in young people, especially when poorly educated, with migraine diagnoses driven by improbably high proportions reporting nausea (44.8%) and vomiting (28.0%) as usual symptoms accompanying their headaches.

**Conclusions:**

Headache is very common in children and adolescents in Ethiopia. This has major public-health implications, since half the country’s population are aged under 18 years.

## Introduction

Over multiple iterations between 2000 and 2019, the Global Burden of Disease (GBD) study has demonstrated that headache disorders are a major cause of global ill health, to the extent of being the second highest cause of disability [1–6]. The data for headache contributing to GBD estimates nevertheless remain deficient: there are still geographical gaps. Further, these data so far have very largely been collected from adults (aged 18–65 years).

A key objective of the Global Campaign against Headache, under the direction of *Lifting The Burden* (LTB), is to quantify headache-attributed burden worldwide [7–10]. In adults, the principal contributors to this are migraine, tension-type headache (TTH), medication-overuse headache (MOH) and the group of other disorders characterized by headache occurring on ≥15 days/month. In children (aged 6–11 years) and adolescents (12–17 years), the same are important, but recent studies have shown, in addition, a prevalent headache disorder characterised by mild pain of short duration (< 1 h) [11, 12]. Termed “undifferentiated headache” (UdH), this is not diagnosable as migraine or TTH but believed to be expressions of these by the immature brain. In these age groups, LTB is conducting a global schools-based programme using a standardised protocol [13] in multiple countries in each of the world’s six major regions, thereby cluster-sampling the world.

This national study in Ethiopia, part of the global programme, is the first to be reported from sub-Saharan Africa (SSA) and the first in a low-income country. Half of Ethiopia’s population are aged under 18 years [14]. While no published data exist for child and adolescent headache in this country, a population-based national survey on adult headache disorders showed that they were common: 1-year prevalence estimates were 17.7% for migraine (higher than the global mean [6]), 20.6% for TTH, 0.7% for probable MOH (pMOH) and 2.5% for other headache on ≥15 days/month [15].

This study estimated the prevalence and attributed burden of headache disorders in children and adolescents in Ethiopia. The purposes were to add to knowledge of the global burden of headache, and to inform local health and educational policies. This paper reports prevalence; a later paper will report on burden.

## Methods

The study, a cross-sectional survey, followed the generic protocol for the global programme [13]. It was conducted in schools selected to be nationally representative. Enquiry was by self-completed questionnaire administered under supervision to pupils within their school classes.

### Ethics and approvals

Approval was obtained from the Institutional Review Board (IRB) of the College of Health Sciences of Addis Ababa University. Subsequently, letters of permission, specifying each school that we planned to survey, were obtained from all four regional education bureaux.

School principals and teachers at each selected school were invited, and agreed, to participate. Information sheets describing the nature and purposes of the survey, and consent forms, were distributed to pupils in the participating schools on the day preceding the survey, and prior consent obtained from or on behalf of each participating child or adolescent (in most sites, teachers signed to signify the consent of children rather than the children themselves, in accordance with the terms of ethics approval).

Data were collected anonymously, and held and used in compliance with data protection legislation.

### Sampling and recruitment

We randomly selected six schools located in Addis Ababa city (two urban schools), Hawassa, Sidama zone, in Southern Nations, Nationalities, and Peoples’ region (one semi-rural school), Asella, Arsi zone, in Oromia region (one semi-rural school) and Bahir Dar, West Gojjam zone, in Amhara region (one urban, one rural school). These were purposively chosen to capture the main cultural, geographical and socioeconomic diversities of the country.

In each school, all classes including pupils aged 6–11 years and/or 12–17 years were invited to participate. In each of these classes, all pupils present on the day were invited to complete the survey instrument, excluding only children for whom parental consent was required but had not been given. The latter, along with pupils of any age unwilling on their own account to take part for any reason, were counted as non-participants. Pupils who happened to be absent from school on the survey day were not part of the sample, and not, therefore, counted as non-participants.

The study was conducted during one academic term, avoiding examination periods, in 2018.

### Numbers

Our target was *N* = 2400, consisting of 200 evaluable participants of each year of age in the range 6–17, drawn from the participating schools in proportions according to their size. Published recommendations suggest larger samples provide limited gain for the increased investment of resources [16].

### Survey instrument

We used the child and adolescent versions of LTB’s Headache-Attributed Restriction, Disability, Social Handicap and Impaired Participation (HARDSHIP) structured questionnaire [13], a modular instrument incorporating demographic enquiry, headache screening and diagnostic questions based on ICHD-3 beta criteria [17] and enquiries into headache-attributed burden. We translated these into Oromiffa (Afan Oromo) and Amharic languages, the two languages most commonly used in Ethiopia, following LTB’s translation protocol for lay documents [18]. The timeframes of enquiry were the preceding 4 weeks (28 days) and 1 week (7 days), except for the module asking specifically about headache yesterday (HY).

These questionnaires were administered to the pupils in their classes, who completed them anonymously under the supervision of an investigator and teacher. After an initial introduction, adolescents, and children competent in reading, completed their questionnaires independently, without further assistance. Younger children were assisted when necessary. Monitoring prevented any pupil copying the responses of another.

Additional questionnaires addressed to teachers and/or principals at each school enquired into relevant school variables [13].

### Data management and entry

Completed questionnaires were removed to Addis Ababa University, and held securely. We performed independent double data-entry into an electronic database, with reconciliation of discrepancies by comparison with source data.

### Analysis

Analyses were performed at University of Mersin, Turkey.

We categorized schools by locality (urban, semi-rural, rural), by pupils’-home income (estimating proportions of pupils coming from low-income homes [less than one quarter, one quarter to one half, half or more than half, categories referred to for simplicity as “higher-middle income”, “middle income”, “lower-middle income” and “low income”]), by teachers’ assessment of school-area income (categorised as “higher-middle”, “lower-middle” or “low”), and by home proximity (estimating proportions of pupils travelling for ≥1 h/day to attend [less than one quarter or one quarter to one half, categorised as “mostly close” and “many distant”]). We used descriptive statistics to present means and standard deviations (SDs) of continuous variables and proportions (%) with 95% confidence intervals (CIs) of categorical data. We used Student’s t-test and Fisher’s exact or chi-squared tests to evaluate differences between groups.

Diagnoses were made during analysis, algorithmically according to HARDSHIP methodology [13, 19]. We first identified headache on ≥15 days/month (reported as headache on ≥14 days/4 weeks), and categorised it according to medication use as probable MOH (pMOH) if reported on ≥14 days/4 weeks or “other headache on ≥15 days/month” if less. To all other reported headaches, the algorithm then applied criteria for UdH (mild intensity and usual duration < 1 h [11]) and finally the ICHD-3 beta criteria [17] for definite migraine, definite TTH, probable migraine and probable TTH, in this order [19]. Remaining cases were unclassified.

We estimated prevalence of each headache type as proportions (%) with 95% CIs, and adjusted observed values for gender and age using official population statistics for Ethiopia [20]. In these analyses, we combined definite and probable migraine and definite and probable TTH [19]. We used chi-squared to compare differences between groups. We used bivariate analysis with odds ratios (ORs) to indicate associations with demographic variables, then the binary logistic regression model, entering gender, age group, school locality and pupils’ home income category for adjusted ORs (AORs).

We calculated proportions reporting HY. We also calculated mean reported headache frequencies (days/week and days/4 weeks) from the responses to two questions: “On how many days in the last week did you have a headache?” and “On how many days in the last 4 weeks did you have a headache?” From these, we predicted 1-day prevalence of headache to compare with the empirical estimates of HY.

In all analyses, we considered *p* < 0.05 to be significant.

## Results

Class lists indicated a total population of 2403, but 54 pupils (male 31 [2.6%], female 23 [1.9%]) were absent on the days of survey. All 2349 who were present agreed to participate, but five (0.2%) were excluded (non-participation proportion = 0.2%) because of incomplete responses. Therefore, the surveyed sample were *N* = 2344 (male 1157 [49.4%], female 1187 [50.6%]). Children (*n* = 1011 [43.1%]) were under-sampled in comparison with adolescents (*n* = 1333 [56.9%]). Overall mean age was 12.0 ± 2.2 years (range: 6–17; median 12).

School variables (Table 1) were clearly influenced by, but did not entirely reflect, Ethiopia’s urban/rural divide (21:79 [21]). A third (36%) of all participating pupils attended urban schools, while the remainder were at schools in semi-rural or rural locations. The majority (59%) were at schools rated lower-middle or low income, as defined above. Teachers rated school-area income similarly: 55% of pupils were from lower-middle- or low-income areas. Most pupils (62%) were at schools close to their homes (fewer than 25% travelling for > 1 h/day).

**Table 1:** School variables (from teachers’ questionnaires), and numbers of participant pupils affected (*N* = 2344)

Variable	Pupils affected	
	n	%
School locality:		
Urban	849	36.2
Semi-rural	1173	50.0
Rural	322	13.8
Pupils’ home-income^a^:		
“Higher-middle”	359	15.3
“Middle”	604	25.8
“Lower-middle”	573	24.4
“Low”	808	34.5
School-area income^a^:		
“Higher-middle”	1059	45.2
“Lower-middle”	926	39.5
“Low”	359	15.3
Home proximity^a^:		
“Mostly close”	1453	62.0
“Many distant”	891	38.0

^a^see text for explanation

### Headache

Headache ever was reported by 1822 participants (observed lifetime prevalence = 77.7%). Table 2 shows one-year prevalence estimates, overall, by headache type and adjusted for gender and age. Almost three quarters (73.6%) reported headache in the preceding year, with migraine the most common type (38.4%: definite 16.0%; probable 22.4%), followed by TTH (20.3%: definite 12.6%; probable 7.7%). Adjusted 1-year prevalence estimates were 72.8% for all headache, 38.6% for migraine, 19.9% for TTH, 12.3% for UdH and 1.2% for all headache on ≥15 days/month. Only four cases (0.2%) were reported of pMOH. There were 22 cases (0.9%) remaining unclassified.

**Table 2:** Crude (observed) 1-year prevalences (% [95% CIs]) of all headache and each headache type, overall and according to demographic variables, and gender- and age-adjusted prevalences

	All headache (*n* = 1726)	Migraine (*n* = 900)	TTH (*n* = 477)	pMOH (*n* = 4)	Other headache on ≥ 15 d/m (*n* = 25)	UdH (*n* = 298)
**Observed prevalences** (% [95% CI])						
Overall (*N* = 2344)	73.7 [71.9–75.5]	38.4 [36.4–40.4]	20.3 [18.7–21.9]	0.2 [< 0.1–0.4]	1.1 [0.7–1.5]	12.7 [11.4–14.0]
Gender						
male (*n* = 1157)	71.0 [68.4–73.6]	36.2 [33.4–39.0]	20.0 [17.7–22.3]	0.2 [0.0–0.5]	1.0 [0.4–1.6]	12.6 [10.7–14.5]
female (*n* = 1187)	76.2 [73.8–78.6]	40.5 [37.7–43.3]	20.7 [18.4–23.0]	0.2 [0.0–0.5]	1.1 [0.5–1.7]	12.8 [10.9–14.7]
Age group (years)						
6–11 (*n* = 1011)	68.4 [65.5–71.3]	40.0 [37.0–43.0]	17.1 [14.9–19.4]	0.1 [0.0–0.3]	0.8 [0.3–1.4]	10.1 [8.2–12.0]
12–17 (*n* = 1333)	77.6 [75.4–80.0]	37.2 [34.6–38.0]	22.8 [20.6–25.1]	0.2 [0.0–0.4]	1.3 [0.7–1.9]	14.7 [12.8–16.6]
Pupils’ home-income^a^						
higher-middle (*n* = 359)	86.1 [82.5–90.0]	46.8 [41.6–52.0]	21.2 [17.0–25.4]	0.3 [0.0–0.9]	1.1 [0.0–2.2]	14.8 [11.1–18.5]
middle (*n* = 604)	73.3 [69.8–76.8]	31.6 [27.9–35.3]	23.8 [20.4–27.2]	0.5 [0.0–1.1]	1.3 [0.4–2.2]	15.2 [12.3–18.1]
lower-middle (*n* = 573)	75.7 [72.2–79.2]	40.8 [36.8–44.8]	21.1 [17.8–24.4]	0.0 [0.0–0.0]	1.2 [0.3–2.1]	11.3 [8.7–13.9]
low (*n* = 808)	66.8 [63.6–70.1]	38.0 [34.7–41.4]	16.8 [14.2–19.4]	0.0 [0.0–0.0]	0.7 [0.1–1.3]	10.9 [8.8–13.0]
School locality						
urban (*n* = 849)	79.6 [76.9–82.3]	43.3 [40.0–46.6]	21.2 [18.5–24.0]	0.1 [0.0–0.3]	1.2 [0.5–1.9]	12.7 [10.5–14.9]
semi-rural (*n* = 1173)	71.1 [68.5–73.7]	35.3 [32.6–38.0]	20.4 [18.1–22.7]	0.3 [0.0–0.6]	1.2 [0.6–1.8]	13.4 [11.5–15.3]
rural (*n* = 322)	67.1 [62.0–72.2]	36.6 [31.3–41.9]	18.0 [13.8–22.2]	0.0 [0.0–0.0]	0.3 [0.0–0.9]	10.2 [6.9–13.5]
**Gender- and age-adjusted prevalences** (% [95% CI])						
Overall (*N* = 2344)	72.8 [71.0–74.6]	38.6 [36.6–40.6]	19.9 [18.3–21.5]	0.2 [< 0.1–0.4]	1.0 [0.6–1.4]	12.3 [11.0–13.6]

*CI* confidence interval, *TTH* tension-type headache, *pMOH* probable medication-overuse headache, *d/m* days/month, *UdH* undifferentiated headache; ^a^ see text or Table 1 for explanation. Odds ratios for associations with demographic variables are in Table 4, and adjusted odds ratios in Table 5

Because the estimate of migraine prevalence was high, we analysed responses to questions likely to drive this diagnosis – essentially those enquiring into the associated features of migraine specified in ICHD, all answered yes/no in our enquiry. Table 3 provides the frequencies of positive responses. Most notable among these was the almost 80% affirmative response to enquiry into phonophobia. Nausea and vomiting – specific to migraine – were reported by 44.8% and 28.0% of all participants with headache.

**Table 3:** Frequencies of positive responses among participants with headache (*n* = 1726) to questions contributing to a diagnosis of migraine

Question (English language version)	Yes (%)
Does exercise (like walking or climbing stairs) make your headache worse?	1049 (60.8)
Do you avoid exercise (like walking or climbing stairs) when you have a headache?	843 (48.8)
With your headache, do you usually feel sick (as though you may throw up)?	773 (44.8)
With your headache, are you usually actually sick (do you throw up)?	484 (28.0)
When you have a headache, do you prefer to be in the dark?	654 (37.9)
When you have a headache, do you prefer to be in the quiet?	1362 (78.9)

### Demographic associations

While headache overall was more prevalent among females (76.2%) than males (71.0%; *p* = 0.0037 [chi-squared]; Table 2), this was reflected only in migraine among the headache types (Table 4). Headache overall was more prevalent among adolescents (77.6%) than children (68.4%; *p* < 0.0001 [chi-squared]), which was reflected in all headache types except migraine. UdH represented 14.8% of all reported headache in children and 18.9% in adolescents, but these numbers mask a fluctuating relationship with age. In a year-by-year analysis, prevalence of UdH rose from 5.9% at age 7 to 14.8% at age 9, fell to 8.6% at age 10, then increased again, steadily, to 18.1% at 14; from 15 to 18 years, it declined rapidly to 3.9%.

**Table 4:** Bivariate analyses of associations of headache types with demographic variables (*N* = 2344)

Variable	Migraine	Tension-type headache	All headache on ≥ 15 d/m	Undifferentiated headache
	Odds ratio [95% CI]			
Gender:				
Male (*n* = 1157)	reference	reference	reference	reference
Female (*n* = 1187)	**1.2** [1.02–1.4]^**1**^	1.05 [0.9–1.3]	1.06 [0.5–2.2]	1.0 [0.8–1.3]
Age group (years)				
6–11 (*n* = 1011)	reference	reference	reference	reference
12–17 (*n* = 1333)	0.9 [0.8–1.05]	**1.4** [1.2–1.8]^**3**^	1.7 [0.8–3.7]	**1.5** [1.2–2.0]^**3**^
Pupils’ home-income^a^				
Higher-middle or middle (*n* = 963)	0.9 [0.8–1.1]	**1.3** [1.1–1.6]^**1**^	1.8 [0.9–3.7]	**1.4** [1.1–1.8]^**2**^
Lower-middle or low (*n* = 1381)	reference	reference	reference	reference
School locality				
Urban (*n* = 849)	**1.4** [1.2–1.6]^**3**^	1.1 [0.9–1.3]	1.1 [0.5–2.3]	1.0 [0.8–1.3]
Semi-rural or rural (*n* = 1495)	reference	reference	reference	reference

*d/m* days/month; ^a^see text or Table 1 for explanation; significant values are emboldened (^1^ *p* < 0.05; ^2^ *p* < 0.01; ^3^ *p* < 0.001)

The influences of income were inconsistent: although, generally, more headache was reported in schools in the higher pupils’ home-income categories, this was true for migraine only in the highest category (Tables 2, 4 and 5). In this same category, all other headache types were reported less frequently than in the category below, although these differences were not significant (Table 2). All headache was more common in urban schools (Table 2), reflected principally in migraine (Tables 4 and 5).

**Table 5:** Binary logistic regression analysis of associations of headache types with demographic variables (*N* = 2344)

Variable	Migraine	Tension-type headache	All headache on ≥ 15 d/m	Undifferentiated headache
	Adjusted odds ratio^**a**^ [95% CI]			
Gender:				
Male (*n* = 1157)	reference	reference	reference	reference
Female (*n* = 1187)	**1.2** [1.01–1.4]^**1**^	1.1 [0.9–1.3]	1.1 [0.5–2.3]	1.0 [0.8–1.3]
Age group (years)				
6–11 (*n* = 1011)	reference	reference	reference	reference
12–17 (*n* = 1333)	0.9 [0.8–1.1]	**1.4** [1.5–1.7]^**2**^	1.6 [0.7–3.7]	**1.5** [1.2–1.9]^**2**^
Pupils’ home-income^b^				
Higher-middle or middle (*n* = 963)	0.9 [0.8–1.1]	**1.3** [1.03–1.55]^**1**^	1.7 [0.8–3.6]	**1.4** [1.07–1.8]^**1**^
Lower-middle or low (*n* = 1381)	reference	reference	reference	reference
School locality				
Urban (*n* = 849)	**1.4** [1.2–1.6]^**3**^	1.1 [0.9–1.4]	1.1 [0.5–2.5]	1.03 [0.8–1.3]
Semi-rural or rural (*n* = 1495)	reference	reference	reference	reference

*d/m* days/month; ^a^adjusted for all variables listed; ^b^see text or Table 1 for explanation; significant values are emboldened (^1^ *p* < 0.05; ^2^ *p* < 0.01; ^3^ *p* < 0.001)

### Headache yesterday (HY)

HY was reported by 630 (36.5%) of 1725 participants with headache (one did not respond to this question). These were 26.9% of the entire sample. Table 6 shows the numbers and proportions by gender, age and headache type. Females reported HY more than males (*p* < 0.001). Adolescents reported only marginally more HY than children, although a year-by-year analysis showed a substantial increase (to 42–44%) in those aged 15–17 years (OR: 1.8 [1.4–2.3]; *p* < 0.001). Headache on ≥15 days/month was, as anticipated, the greatest contributor to HY proportionately (by definition, > 50% was expected), but its overall impact was limited by its relatively low prevalence.

**Table 6:** Proportions of those with headache reporting headache yesterday, overall and by age, gender and headache type, and predicted proportions, overall and by headache type

Headache type	Headache yesterday				
	Reported proportion n (%)	Predicted proportion			
		Mean reported headache frequency		Predicted headache yesterday (%)	
		days in last week (F7)	days in last 4 weeks (F28)	calculated as F7/7	calculated as F28/28
Any headache (*n* = 1725)^a^	630 (36.5)	1.5 ± 1.6	2.6 ± 3.2	21.4	9.3
male (*n* = 821)	253 (30.8)	not calculated			
female (*n* = 904)	377 (41.7)				
6–11 years (*n* = 692)	248 (35.8)				
12–17 years (*n* = 1033)	382 (37.0)				
Migraine (*n* = 899)^a^	366 (40.7)	1.6 ± 1.6	2.8 ± 2.9	22.9	10.0
Tension-type headache (*n* = 477)	157 (32.9)	1.2 ± 1.4	2.0 ± 2.3	17.1	7.1
All headache on ≥15 days/month (*n* = 29)	19 (65.5)	4.5 ± 2.3	16.1 ± 2.6	64.3	57.5
Undifferentiated headache (*n* = 320)	86 (26.9)	1.0 ± 1.3	1.8 ± 2.3	14.3	6.4

^a^one participant did not respond to this question

Table 6 also shows mean reported headache frequencies based on the two related questions (see Methods). HY for all headache was reported more commonly than predicted, the discrepancy being much greater when prediction was based on the 4-week enquiry. This discrepancy was evident in migraine, TTH and UdH, but, for headache on ≥15 days/month, HY was approximately matched by both predictions.

## Discussion

This was the first study to be reported from SSA within the schools-based enquiry into child and adolescent headache, conducted as a major fact-finding component of the Global Campaign against Headache [7, 8]. The key finding was that headache was prevalent among these age groups, affecting almost three quarters. Migraine was the most often reported type (over one third), followed by TTH (one fifth); UdH, while common at 12.7%, was less so than these specific headache disorders. Headache on ≥15 days/month was reported by 1.3%, very little of this being pMOH (0.2%).

Associations with gender were much as expected but, rather surprisingly, increased prevalence of migraine was not seen among adolescents. In this context we need to say more about UdH. This recently characterised disorder is understood to represent expressions of migraine or TTH by the immature brain [11]. Accordingly, its prevalence as a proportion of all reported headache is expected to decline with increasing age, as seen in Turkey [11] and Austria [12]. In Ethiopia the picture was more complex, but what was clear was that the prevalence of UdH fell sharply after age 14 years as other headache disorders increased. Presumably this change reflected rapid brain development after puberty, but age-related decline in UdH was expected earlier. UdH has been defined as short-duration mild headache, with duration somewhat arbitrarily fixed at < 1 h [11], while children’s sense of time is unreliable. Although we are not presenting headache duration here, reported duration for 64.9% of all headache, including 84.4% of cases diagnosed as probable migraine, was < 2 h.

In relation to this, we must question our finding of 38.6% migraine prevalence, which we acknowledge to be implausible. LTB’s study of adult headache disorders in Ethiopia estimated 1-year prevalence of migraine at 17.7% (with rather more TTH, at 20.6%) [15]. We do not believe that migraine is more than twice as common in these younger age-groups. In children in particular, headache diagnosis is notoriously difficult, dependent as it is on comprehension of language on the one hand (formulation of ICHD criteria into questions that are clear and unambiguous to children is a challenge) and subjective evaluations on the other (over a quarter [26.5%] of cases with reported headache duration of < 1 h were diagnosed as probable migraine, the distinction from UdH resting solely on reported headache intensity). Questions relating to associated symptoms – likely to drive diagnosis – are especially difficult to structure other than as leading questions, with yes/no response options. Although these questions had been successfully tested elsewhere [11–13], children in particular may be susceptible to suggestion. We saw this, probably, in the almost 80% affirmative response to phonophobia (Table 3), which deprived this symptom of diagnostic utility (although the much lower photophobia response [37.9%] diminished its impact, since both must be present for either to contribute to migraine diagnosis [17]). The responses to questions on nausea (44.8% affirmative) and vomiting (28.0% affirmative), although lower, were probably more influential, since these symptoms are specific to migraine. If it were true that over one quarter of all participants with headache usually vomited during headache episodes, and nearly half were nauseated, then the proportion with migraine was undoubtedly high. But we doubt that it was true.

The veracity of our participants’ responses is questionable again in the over-reporting of HY, at least by those with episodic headache. Headache frequencies reported over the preceding 7 and 28 days were perfectly feasible for all headache types, but, other than in those with headache on ≥15 days/month, HY claims were not: approximately double the predictions based on the former and four times the predictions based on the latter. HY reporting should be free from recall error: it is from this, essentially, that it derives its value as a measure [16]. One interpretation, therefore, is that HY reporting was correct, while accuracy diminished as memory faded over 7 then 28 days. But we doubt this also: it is implausible that more than one quarter of these young participants had headache on the day in question (which was, presumably, no different from any other day). Were they dissembling? We doubt this too, but, if the explanation lies elsewhere, we do not know what it is. No problems were encountered with the same question in adults [22].

In future surveys of children and adolescents, some questions may need to be reformulated, even though they were not problematic in Turkey or Austria [11–13]. However, as this series of studies is continued elsewhere in the world, it may transpire that epidemiological diagnosis of headache type in these age groups is an inherently inexact process. This would not be surprising, if our belief is correct that headache is evolving from an undifferentiated form. Neither would it be a revelation: many past surveys have failed to diagnose large proportions of reported headaches, often without comment (for discussion of this, see [11]).

While, therefore, some uncertainty surrounded headache type, prevalence of headache overall (1-year: 72.8%) was the same as was found in children and adolescents in Turkey (73.7%) with the same questionnaire [11]. The Austrian study of adolescents (10–18 years), also using the same questionnaire, reported a 1-year headache prevalence of 75.7% [12], not different from our 77.6% in adolescents (12–17 years). These values appear robust, and leave no doubt that, at least in terms of prevalence, headache is no less a problem in these age groups than in adults. It is interesting to compare our data with the adult data from Ethiopia: 1-year prevalence of headache was 44.9%, with a female preponderance of 4:3, of migraine 17.7%, with a female preponderance of 3:2, and of TTH 20.6%, with marginal female preponderance [15]. The difference between these is in migraine only, although there is the diagnostic *caveat*. These two datasets indicate that not all childhood headache survives into adulthood, and what may appear as migraine in children may not persist as migraine in adults. This is somewhat contrary to received wisdom, raising a query for future studies.

The strengths of this study lie in its adequate sample size, the very high participation proportion and the tested and validated methodology [11–13], although the last proved imperfect in Ethiopia in one important respect. We have already acknowledged this limitation, which has no easy solution. Whether parents might give a better account of their children’s symptoms than the children themselves is very questionable: clinical experience rather suggests the opposite. In any case, on a general level, parental questioning requires a quite different protocol; in this highly rural country characterised by low income and poor education, it would have been impractical. The questionnaire could not be validated in the languages used in Ethiopia for the same reason as in the adult study [15]: with very few headache experts in the country, validation would have required at least one to take time out from the health service in order to re-interview a subset of participants in the field, and it could not be done. A third limitation was that we surveyed in only two of more than 80 active languages in Ethiopia [23]. Respecting the pupils’ preference, we used the local language(s) of each school: Amharic in Addis Ababa, Hawassa and Bahir Dar; Oromiffa and Amharic (the latter preferred by most adolescents) in Asella. In fact these are the most widely spoken languages in Ethiopia – Oromiffa by 33.8% and Amharic (the national working language) by 29.3% of the total population according to 2007 census data [23]. Nevertheless, our survey was, on this account, arguably representative of only a majority (63%) of the population. The additional resources required to add just one language could not be justified when the next most common (Somali) is used by only 6.2% [23]. Finally, schools-based sampling is valid when education is compulsory and uptake is high [16]. We acknowledge a further, possibly important, limitation in this regard. Primary education in Ethiopia is, officially, from 7 to 14 years of age (grades 1–8), and secondary education from 15 to 18 years (grades 9–12). According to Ministry of Education data for 2018/19, school intake among those aged 7 years exceeded 90% nationally (96.5% among males and 88.2% among females), with a higher *apparent* intake rate of 136.6% reflecting enrolment in grade 1 of many younger or older children [24]. But high drop-out rates from grade 2 onwards greatly reduce transition to secondary education (nationally, 32.0% in 2018/19: 48.5% for grades 9–10 and only 14.8% for grades 11–12 [25]). In a low-income country, economic factors weigh heavily: for many, even basic educational expenses may be unaffordable, while parents may depend on their children’s labour at home or for income generation [26]. Children lost to our study were probably, largely, from the poorest homes, with Table 2 suggesting some bias might result, but no other sampling method is likely to obviate this.

## Conclusions

Headache is very common in children and adolescents in Ethiopia – apparently more so than in adults – a finding with major public-health implications for a country in which half the population are aged under 18 years [14]. With no similar study yet from elsewhere in the whole of SSA, or from another low-income country, this finding also contributes to knowledge and understanding of child and adolescent headache globally.

## Data Availability

The data are held on file at University of Mersin. Once analysis and publications are completed, they will be freely available for non-commercial purposes to any person requesting access in accordance with the general policy of the Global Campaign against Headache.
